# Two Berberis Cases

**Published:** 1883-02

**Authors:** E. Rushmore

**Affiliations:** Plainfield, N. J.


### TWO BERBERIS CASES.

E. Rushmore, M. D., Plainfield, N. J.

The December number of this journal contains a report of cases treated with Berberis. They called to mind two cases in which it apparently acted with promptness and efficiency, and which I believe may be worth reporting.

Case I. Mr. P., about thirty-five years of age, has been subject for several months to frequent attacks of severe pain in the right lower dorsal region, which he describes as spasmodic, colicky, aching, drawing. The pain seems to extend from the back to the stomach, or *vice versa*, and to go around the right side to the abdomen and toward the bladder, but never seems to reach the bladder. Sometimes it seems to extend to the left side of the back. The attacks begin mostly in the latter part of the day—never at night; they are attended with tenderness of the back, often sour, acrid vomiting, which relieves; chilliness or heat, and greatly increased urinary secretion. Cathartics and lying on something hard have sometimes mitigated the attacks. He has a bitter mouth in the morning and a feeling of general debility.

He received one dose of Berberis vulgaris$^{40m}$ (Fincke) dry, on the tongue. He lived far from me and I did not see him again. Nineteen days later he wrote he "would not have believed it possible to feel as much better as he does." He has only a little lameness in the back for a short time in the morning—no more colic or back-aches. Appetite better. Two weeks after this he received a single beneficial dose of a high potency for symptoms of disturbed, unrefreshing sleep. About eight weeks after the dose of Berberis was given he had his first and last attack of pain. It was attended with perspiration, and he received one dose of Veratrum album$^{cm}$. I heard directly from him eight months after this attack and he was well and had had no more.

Case II. Mrs. P., æt. 57, reports, December 2d, 1881, that she has suffered for nine months from pain under the finger-nails, with coldness of the feet, extending above the ankles, and swelling of some of the finger-joints. At that time the only remedy under "pain under the finger-nails" in *Lippe's Repertory* was Berberis. (I have since added Bismuth from the *Guiding Symptoms*.) She received Berberis$^{900}$ (Fincke), six powders, to take one every evening and morning. Eleven days later she reported improvement and got no medicine.

January 30th, 1882, writes: "There has been a slight return of pain under the finger-nails for two or three days."

She then got Berberis $^{40m}$, seven powders, to take one every morning till better, but was again relieved before she had taken all.

Three months later applied for treatment for some other symptoms. There has been no further mention of pain under the nails.

